# RNA-Seq improves annotation of protein-coding genes in the cucumber genome

**DOI:** 10.1186/1471-2164-12-540

**Published:** 2011-11-02

**Authors:** Zhen Li, Zhonghua Zhang, Pengcheng Yan, Sanwen Huang, Zhangjun Fei, Kui Lin

**Affiliations:** 1 College of Life Sciences, Beijing Normal University, 19 Xinjiekouwai Street, Beijing, 100875, China; 2 Key Laboratory of Horticultural Crops Genetic Improvement of Ministry of Agriculture, Sino-Dutch Joint Lab of Horticultural Genomics Technology, Institute of Vegetables and Flowers, Chinese Academy of Agricultural Sciences, 12 Zhongguancunnan Street, Beijing, 100081, China; 3 Boyce Thompson Institute and USDA Robert W. Holley Center for Agriculture and Health, Cornell University, Tower Road Ithaca, New York, 14853-1801, USA

## Abstract

**Background:**

As more and more genomes are sequenced, genome annotation becomes increasingly important in bridging the gap between sequence and biology. Gene prediction, which is at the center of genome annotation, usually integrates various resources to compute consensus gene structures. However, many newly sequenced genomes have limited resources for gene predictions. In an effort to create high-quality gene models of the cucumber genome (*Cucumis sativus *var. *sativus*), based on the EVidenceModeler gene prediction pipeline, we incorporated the massively parallel complementary DNA sequencing (RNA-Seq) reads of 10 cucumber tissues into EVidenceModeler. We applied the new pipeline to the reassembled cucumber genome and included a comparison between our predicted protein-coding gene sets and a published set.

**Results:**

The reassembled cucumber genome, annotated with RNA-Seq reads from 10 tissues, has 23, 248 identified protein-coding genes. Compared with the published prediction in 2009, approximately 8, 700 genes reveal structural modifications and 5, 285 genes only appear in the reassembled cucumber genome. All the related results, including genome sequence and annotations, are available at http://cmb.bnu.edu.cn/Cucumis_sativus_v20/.

**Conclusions:**

We conclude that RNA-Seq greatly improves the accuracy of prediction of protein-coding genes in the reassembled cucumber genome. The comparison between the two gene sets also suggests that it is feasible to use RNA-Seq reads to annotate newly sequenced or less-studied genomes.

## Background

As new sequencing technologies develop, thousands of eukaryotic genomes across all kingdoms of life will be sequenced during the next decade [1,2], and this trend will spark an improvement in our knowledge of evolutionary biology and functional genomics. Genome annotation is a stepping stone to bridge the gap between genomic sequences and the biology of organisms [3]. It can be stated that the quality of genome annotations represents the value of genome sequences.

Gene prediction, within the process of genome annotation, is a complex endeavor. In eukaryotic species, it is usually carried out by integrating multiple sources of evidence [4], such as complementary DNA (cDNA), proteins in closely related species, and *de novo *predictions [5]. Representing the integral sequences of messenger RNAs (mRNAs), full-length cDNAs (FL-cDNAs) are recognized as the gold-standards for discovering and annotating gene structures in eukaryotic genomes [5,6]. Additionally, even incomplete cDNAs, i.e. expressed sequence tags (ESTs), provide more accurate evidence than other sources. Nevertheless, until recently, the sequencing of cDNA was a laborious and capital-intensive task.

Thanks to the massively parallel cDNA sequencing (RNA-Seq) technologies [7], scientists can obtain cDNA fragments from transcriptomes with reasonably complete coverage in a reduced time scale and at a lower cost [8]. With its informative content, RNA-Seq is expected to revolutionize the prediction of genes [9]. RNA-Seq has been used to improve the genome annotations, including: (i) correcting predicted gene structures [10]; (ii) detecting new alternative splicing isoforms [11]; and (iii) discovering new genes and new transcripts [12,13]. However, most of these applications focused on species with well-annotated genomes, such as human, mouse, yeast, *Arabidopsis thaliana*, and rice. Among these studies, Trapnell, Williams and Pertea *et al*. and Guttman, Garver and Levin *et al*. correctly reconstructed full-length transcripts for most known expressed genes in specific mouse tissues [12,13]; nevertheless, their procedures still need to be tested in other eukaryotic genomes, because of varied genome characteristics [8]. For less-studied genomes, Denoeud, Aury and Da Silva *et al*. used the short RNA-Seq reads to build thousands of gene models for the grape genome [14]; however, fewer genes were predicted than in the public annotation [15].

Although far from perfect, the considerable potential demonstrated in these studies for the applicability of RNA-Seq in gene predictions encourages us to update the original gene prediction of the cucumber genome (*Cucumis sativus *var. *sativus *line 9930), which was annotated and published in 2009 [16]. Therefore, based on EVidenceModeler (EVM) [17], we built a genome annotation pipeline in which we incorporated analyses of Solexa/Illumina RNA-Seq reads. In an attempt to provide a high-quality gene set for the scientific community and for further study, we reassembled and reannotated the cucumber genome. We subsequently compared the two versions of the gene predictions to evaluate any improvements brought about by RNA-Seq. The comparison presented here supports the hypothesis that RNA-Seq has a positive impact on gene prediction of the cucumber genome.

## Results and Discussion

### Genome reassembly

Using the improved SOAPdenovo program [18] (Release 1.04), we reassembled the cucumber genome by integrating additional large insert paired-end Illumina GA reads from *Cucumis sativus *var. *sativus *(7.4-fold genome coverage, 5 Kb insert size) and from *Cucumis. sativus *var. *hardwickii *(3.8-fold, 5 Kb insert size; 3.2-fold, 10 Kb insert size; see Additional file 1, Table S1 for details). The final assembly (assemVer 2.0) spans 197 Mb and contains 12, 845 scaffolds (see Additional file 1, Table S2 for details). This is approximately 46 Mb less than the previous assembly (assemVer 1.0) and this difference mostly represents redundant repetitive sequences and contaminating sequences. The N50 and N90 contig sizes of assemVer 2.0 are 37.9 Kb and 8.9 Kb, respectively, and 90% of the assembly falls into 153 scaffolds larger than 281 Kb. Compared to assemVer 1.0, assemVer 2.0 is more contiguous, thus facilitating genome annotation.

### Reconstructing transcripts from RNA-Seq by *de novo *assembly and 'align-then-assemble' approaches

We obtained about 220 million Solexa/Illumina RNA-Seq reads from poly(A) RNAs extracted from 10 cucumber tissues (Table 1).

**Table 1:** Number of *de novo *assemblies and "align-then-assembled" transcripts.

Tissues	# RNA-Seq reads	# *De novo *assembled transcripts	Mapped reads	# "Align-then-assembled" transcripts
Ovary	19, 247, 768	86, 994	17, 656, 392 (91.7%)	52, 530
Fertilized ovary	18, 466, 067	81, 650	17, 047, 763 (92.3%)	50, 987
Unfertilized ovary	19, 111, 746	84, 628	17, 394, 685 (91.0%)	52, 003
Root	18, 732, 466	86, 572	17, 162, 238 (91.6%)	52, 167
Stem	24, 535, 215	71, 977	22, 789, 659 (92.9%)	45, 710
Leaf	26, 400, 675	79, 344	24, 405, 569 (92.4%)	49, 351
Male flower	26, 050, 858	83, 957	24, 531, 662 (94.2%)	51, 630
Female flower	23, 818, 868	85, 345	21, 886, 487 (91.9%)	51, 701
Tendril	22, 472, 146	71, 489	20, 585, 234 (91.6%)	44, 658
Base part of tendril	21, 653, 855	70, 260	19, 556, 866 (90.3%)	44, 995

Two different approaches, *de novo *assembly and 'align-then-assemble' [8], were used to reconstruct transcripts from these RNA-Seq reads. The *de novo *assembly was carried out by Inchworm, a *de novo *assembler of RNA-Seq in Trinity [19], which reconstructed 802, 216 *de novo *contigs from the 10 tissues (Table 1). We applied CD-HIT [20] to remove some *de novo *contigs, such as assembled artifacts with low-coverage or redundancies from different tissues. Finally, 258, 876 *de novo *contigs assembled by RNA-Seq reads remained for gene prediction. In the 'align-then-assemble' approach, we mapped and generated spliced alignments of the RNA-Seq reads from each tissue to the reassembled cucumber genome using Bowtie [21] and TopHat [22] (Table 1; Additional file 1, Table S3 for mapping details of reads). Cufflinks [13] was then used to reconstruct 220, 590 transcripts belonging to 59, 481 transcriptional units from the alignments of 10 tissues. However, a complete open reading frame (ORF) could be found in only 9, 964 (4.5%) transcripts reconstructed by Cufflinks using *getorf *in EMBOSS [23].

### Reannotation of the cucumber genome

The reassembled cucumber genome (assemVer 2.0) contains 23, 248 protein-coding genes with 25, 600 transcripts (Table 2), 621 tRNAs, 20 rRNA, 157 snRNAs, 201 snoRNAs, 1, 025 miRNAs (Additional file 1, Table S4) and 217, 826 transposable elements (Additional file 1, Table S5). This version of the annotation is labeled as annotVer 2.0 (available at http://cmb.bnu.edu.cn/Cucumis_sativus_v20/).

**Table 2:** Summary statistics and annotation comparison of cucumber genome.

Genome	assemVer 1.0	assemVer 2.0	assemVer 2.0
Size (bp)	243, 568, 484	197, 271, 687	197, 271, 687
GC Content	31.50%	31.86%	31.86%

**Genes**	**annotVer 1.0**	**annotVer 1.0 (mapped)**	**annotVer 2.0**
Number of Genes	26, 682	20, 923	23, 248
Number of Genes on Plus Strand	13, 331	10, 488	11, 656
Number of Genes on Minus Strand	13, 351	10, 435	11, 592
Mean Gene Length (bp)	2, 685	2, 966	3, 213
Gene density (Kb/gene)	9.1	9.4	8.5
Number of Transcripts	26, 682	20, 923	25, 600
Percent of Transcripts with Introns	69.37%	74.36%	81.55%
Mean Transcript Length (bp)	2, 685	2, 966	3, 314
Mean CDS Length	1, 046	1, 095	1, 134
Percent Coding	11.49%	11.64%	14.75%

**Exons**	**annotVer 1.0**	**annotVer 1.0 (mapped)**	**annotVer 2.0**
Number	117, 116	100, 721	136, 008
Mean Number per Transcript	4.39	4.81	5.31
GC Content	44.96%	43.73%	42.03%
Mean Length (bp)	239	228	270
Total Length (bp)	27, 991, 662	22, 988, 520	36, 686, 879

**Introns**	**annotVer 1.0**	**annotVer 1.0 (mapped)**	**annotVer 2.0**
Number	90434	79, 798	110, 408
Mean Number per Transcript	3.39	3.81	4.31
GC Content	32.18%	32.37%	32.44%
Mean Length (bp)	483	490	436
Total Length (bp)	43, 647, 564	39, 074, 873	48, 152, 435

**UTRs**	**annotVer 1.0**	**annotVer 1.0 (mapped)**	**annotVer 2.0**
Number of Genes Having UTRs	NA	NA	18, 690
Mean UTR Length (bp)	NA	NA	234
Number of 5' UTRs	NA	NA	15, 703
Mean 5' UTR Length (bp)	NA	NA	175
Number of 3' UTRs	NA	NA	16, 737
Mean 3' UTR Length (bp)	NA	NA	289.08

Compared with the published annotation of the cucumber genome (labeled as annotVer 1.0, Table 2), annotVer 2.0 contains 3, 434 fewer protein-coding genes, mostly because of the reduced size of the reassembly and the removal of some contaminating bacterial segments implied by about 2, 000 bacterial genes in annotVer 1.0. Consistent with the reduction of gene number in annotVer 2.0, there is an increase in the number of multi-exon genes, which indicates an improvement of the protein-coding prediction to some extent, because the prediction of single-exon genes is still unreliable in eukaryotic genomes.

Two other improvements resulting from the incorporation of RNA-Seq are the prediction of untranslated regions (UTRs) and alternative splicing isoforms. Of the 23, 248 protein-coding genes in annotVer 2.0, 18, 690 genes have UTRs and 1, 935 genes appear to have alternative splicing isoforms. In general, incorporating RNA-Seq reads offers overwhelming evidence for the prediction of these two features. The prediction of UTRs was uncertain before the appearance of RNA-Seq, because of the incompleteness of ESTs and the difficulty of collecting *bona fide *FL-cDNAs. Furthermore, because they are not well conserved across species, comparative predictive techniques are not suited to UTR detection. However, the use of high-throughput RNA-Seq from the same species naturally removes both of these difficulties. Similarly, RNA-Seq provides evidence pointing to the potential for alternative splicing, though it is still quite difficult to determine full-length isoforms from these short reads. With the help of *de novo *assemblies and PASA assemblies [24], 2, 352 full-length isoforms of 1, 935 genes were identified. RNA-Seq provides an opportunity to comprehensively study alternative splicing events in cucumber, as in other species.

### Evidence Support for multi-exon genes

For the 18, 580 multi-exon genes in annotVer 2.0, we inspected different sources of evidence for them, and the results suggested that most of the multi-exon genes were supported by reliable evidence, such as transcript evidence or protein evidence [5]. In fact, there are three sources of evidence in our pipeline: transcript evidence from RNA-Seq or ESTs, proteins from related species, and predictions from *de novo *predictors (see Methods), which actually provided introns in the final gene structures [17]. We did not include the predictions of Augustus [25] and Geneid [26] in this analysis because the two predictors had used RNA-Seq information and homologous proteins, respectively.

**Figure 1:**
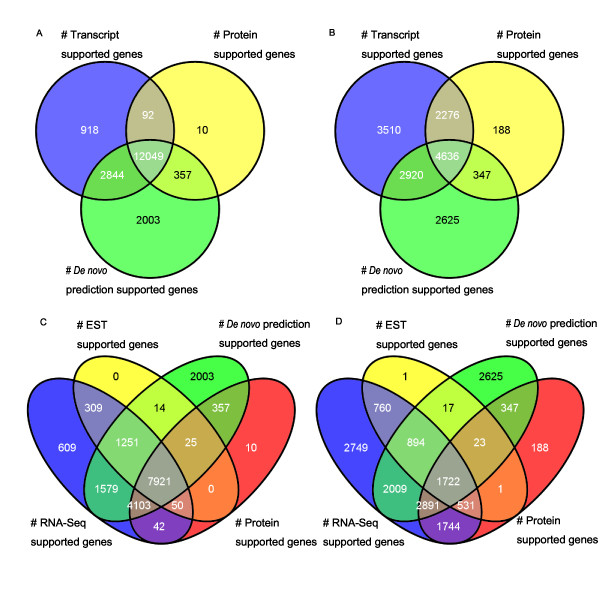
**Venn diagram for sources of evidence for multi-exon gene prediction**. The different colors indicate various sources of evidence, and the numbers are the number of gene models supported by each kinds of evidence. A. Evidence support for predicted genes; B. Evidence support for full-length supported genes; C. Comparison of contributions of RNA-Seq and ESTs to genes; D. Comparison of contributions of RNA-Seq and ESTs to full-length supported genes.

Our analysis shows that most of the multi-exon genes are supported by reliable evidence, such as transcripts or proteins. In Figure 1.A, 16, 270 (87.5%) multi-exon genes are supported either by transcript evidence or protein evidence, while 12, 049 (64.8%) genes are supported by all three kinds of evidence. To check which evidence has a more positive effect on the gene prediction, we traced the sources of evidence for full-length supported genes, because full-length-supports, for single gene prediction, ensure the accuracy of the gene structural prediction. Herein, a gene that is supported by one type of evidence is termed as full-length supported gene if all of the gene's introns are fully supported by the evidence. As expected, transcript evidence from RNA-Seq or ESTs supported more full-length genes than the other two kinds of evidence for multi-exon genes in annotVer 2.0 (Figure 1.B). In fact, 13, 342 (71.8%) multi-exon genes are full-length supported genes when supported by transcript evidence, while 10, 528 (56.7%) genes and 7, 447 (40.1%) genes are fully supported by *de novo *predictions and protein evidence, respectively.

*De novo *predictions are also necessary for gene predictions. Although RNA-Seq has a high coverage, *de novo *predictions actually support more multi-exon genes than do transcripts (Figure 1.A), because of the large number of genes generated by the three *de novo *predictors, for example, GeneMark.hmm-ES predicted more than 40, 000 genes. Furthermore, about one tenth of the multi-exon genes (2, 003/10.8%) are supported only by *de novo *predictions, which indicates that *de novo *predictions are indispensable to the completeness of the final gene sets.

As transcript evidence plays a considerable role in the multi-exon gene prediction, we examined the contributions of RNA-Seq to the final gene set, especially when compared with ESTs. Figure 1.C shows that most of the ESTs are covered by RNA-Seq and none of the multi-exon genes are supported only by ESTs. Despite the fact that one gene in Figure 1.D is full-length supported by one EST, we maintain that RNA-Seq could replace ESTs in the process of protein-coding gene prediction.

Evidential support for multi-exon genes suggests that RNA-Seq has an innate capability for high coverage in protein-coding gene predictions. Transcript evidence is taken as the most valuable evidence in protein-coding gene prediction, as it often identifies exact intronic boundaries [6]. RNA-Seq, among all the transcript evidence that affects gene prediction, is the one that could increase the number of genes supported by transcript evidence and improve the structural predictions. RNA-Seq could also be used in place of ESTs as the major transcript evidence, which liberates scientists from time-consuming work in traditional cDNA sequencing projects. Although RNA-Seq still could not replace the role of FL-cDNA in gene discovery, sophisticated methods of transcript reconstruction through RNA-Seq in the near future may help us to reconstruct more full-length transcripts.

### Improvements of protein-coding gene prediction

**Figure 2:**
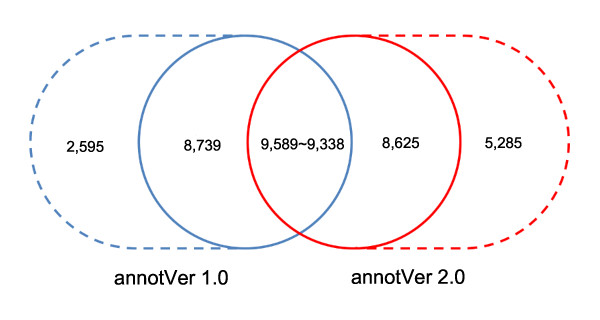
**Venn diagram for protein-coding gene structural changes**. Venn diagram showing the comparative analysis of gene structures (regardless of UTRs). The number in the overlapped region represents the number of genes with the same structures; the two numbers in the circles with the solid lines are the number of genes with the same loci but different structures; the two numbers in the dashed circles are the number of specific genes predicted by annotVer 1.0 and annotVer 2.0 respectively.

The prediction of protein-coding genes has many different features in annotVer 2.0, such as longer transcripts and coding sequences (CDS), more and longer exons, and more and shorter introns (Table 2). To analyze the gene structural differences between annotVer 1.0 and annotVer 2.0, we mapped the CDSs of annotVer 1.0 to the genome, assemVer 2.0, by spaln [27]. Of 23, 216 CDSs having a hit, 20, 923 CDSs matched complete gene structures with start and stop codons. Some of the 20, 923 CDSs mapped to the same gene loci in assemVer 2.0, indicating that some redundancies in the original assembly have been removed in assemVer 2.0 (see Additional file 1, Figure S1 for example). The comparison of gene predictions is illustrated in Figure 2, in which 18, 328 genes in annotVer 1.0 fall into 17, 963 gene loci in annotVer 2.0. The structures of 9, 589 genes in annotVer 1.0 are consistent with the structures of 9, 338 genes in annotVer 2.0. The different number of consistent gene structure in the two versions results from mapping of two or more genes at one locus, as mentioned above. Figure 2 also illustrates 2, 595 genes in annotVer 1.0 and 5, 285 genes in annotVer 2.0 that are located in different loci in the reassembled cucumber genome. To further compare the difference of the two gene sets, we performed four analyses, which all suggest that annotVer 2.0 is better than annotVer 1.0.

**Figure 3:**
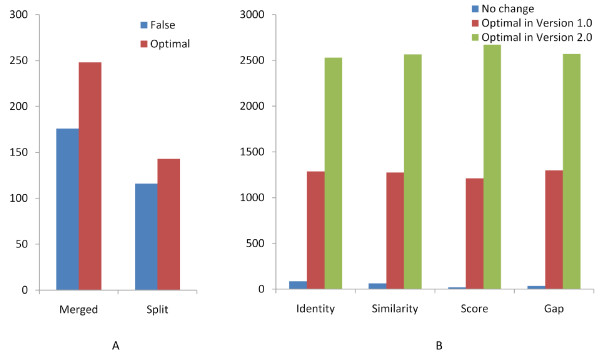
**Structural prediction improvement in annotVer 2.0**. Comparisons of merged/split events and global alignments between genes with different structures in annotVer 1.0 and annotVer 2.0. A. Blue bars, genes falsely merged/split in annotVer 2.0; red bars, genes optimally merged/split in annotVer 2.0. B. Blue bars indicate genes predicted in annotVer 1.0 and annotVer 2.0 giving exactly the same result; red bars indicate genes in annotVer 1.0 that have better statistics than their counterparts in annotVer 2.0; and green bars indicate genes in annotVer 2.0 that have better statistics compared with their counterparts with different structures in annotVer 1.0.

The first analysis concerned merged or split gene structures, which map to the same locus but with different gene numbers in the two versions (Additional file 1, Figure S2). We found that 1, 666 genes in annotVer 1.0 merged into 799 genes in annotVer 2.0, and 750 genes in annotVer 1.0 split into 1, 589 genes in annotVer 2.0. To discriminate between true and false positive merged/split events, we searched each group of genes in one locus against UniProt plant proteins using BLASTP [28]. Each group of genes has one merged gene in one version and several split genes in the other. The number and consistency of hits for the split members decided whether a merged/split event is optimal (see Methods). Figure 3.A illustrates that the number of optimal merged/split events in annotVer 2.0 is greater than in annotVer 1.0.

The second analysis focused on genes at the same locus but with different structures (see Additional file 1, Figure S3 for example). There are 5, 824 pairs of genes, each of which was composed of an annotVer 1.0 and annotVer 2.0 gene that only map to each other, but are structurally different. We then launched a Pfam domain search by InterProScan [29] and performed the global pairwise alignments by *stretcher *in EMBOSS [23] on each pair (see Methods). The search of InterProScan found 1, 817 different kinds of Pfam domains in 4, 297 (73.8%) genes in annotVer 1.0, whereas, 1, 861 different Pfam domains were found in 4, 399 (75.5%) genes in annotVer 2.0. In the same way, when identity, similarity, score, and gaps in alignments are compared, global pairwise alignments also suggests that genes in annotVer 2.0 are more optimal than genes in annotVer 1.0 (Figure 3.B).

In the third analysis, the presence of non-overlapped locus protein-coding genes implies specific genes in different predicted gene sets. To measure the reliability of version specific genes in the two sets, we compared the percentages of BLASTP hits to UniProt plant proteins and multi-exon genes between annotVer 1.0 and annotVer 2.0. The BLASTP results indicated that the prediction of annotVer 2.0 produces more genes and a higher percentage of hits (3, 134, 59.3%) to UniProt than annotVer 1.0 (684, 26.4%). Meanwhile, the specific genes in annotVer 2.0 contain a significantly higher percentage of multi-exon transcripts (4, 385, 77.8%) than those in annotVer 1.0 (1, 216, 46.9%).

**Figure 4:**
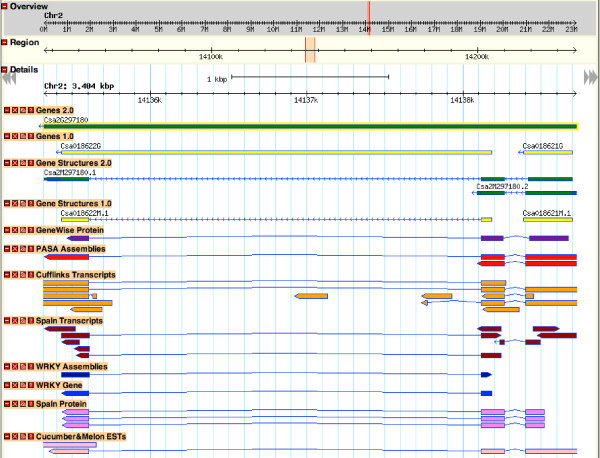
**An example of *WRKY *gene prediction**. An example of *WRKY *gene prediction with and without RNA-Seq reads. Green tracks are the *WRKY *genes predicted in annotVer 2.0. Yellow tracks are the *WRKY *genes predicted in annotVer 1.0. The purple track is the GeneWise alignment of proteins and the red track is shown for the transcripts generated by PASA from *de novo *contigs. The orange track shows the transcripts reconstructed by 'align-then-assemble' approach implemented by Cufflinks and the dark red track illustrates *de novo *contigs and ESTs of cucumber aligned by spaln. The blue track is the FL-cDNA of the cucumber *WRKY *assembly and gene aligned by spaln, and the two pink tracks are proteins and ESTs aligned by spaln.

Finally, a small dataset of 33 *WRKY *genes and 35 *WRKY *gene assemblies generated in an experimental study [30] gave us an opportunity to directly compare the accuracy of the two gene predictions. We aligned the 33 *WRKY *genes and 35 assemblies to the newly assembled genome of cucumber using spaln [27] and mapped out 32 loci, where *WRKY9 *and *WRKY10 *aligned to the same locus due to the change in assembly. The prediction in annotVer 1.0 missed 2 loci of *WRKY *genes and predicted only 22 *WRKY *genes with the same structures as the experimental data. By contrast, annotVer 2.0 only missed 1 *WRKY *gene and 26 of them had structures consistent with the experimental data. Even though five *WRKY *gene structures in annotVer 2.0 are different from the experimental data, one of them, which is supported by protein evidence, shows a better structure than the experimental data (Figure 4). In this example, the experimental data is consistent with annotVer 1.0, but the transcripts reconstructed by RNA-Seq, ESTs and homologous proteins all indicate an extra exon at the 5' end, whereas a single-exon gene is predicted by annotVer 1.0. Although the first exon boundary is different in the two isoforms, they are supported either by GeneWise or transcript evidence. Thus in this case, two genes in annotVer 1.0 are merged into one gene with two isoforms when predicted in annotVer 2.0.

In all four analyses, annotVer 2.0 shows a better performance than annotVer 1.0. Although the processes of generation of the two gene sets are different (Glean for annotVer 1.0 [16]), they are comparable, because the principles of evidence combination are nearly the same. In fact, annotVer 2.0 used less protein evidence and homolog EST evidence than did annotVer 1.0 [16]; however, using RNA-Seq compensates for this minor deficiency and actually obtains a better gene set. Thus, adopting the RNA-Seq technique proved to be vital to the quality of protein-coding gene prediction in the reassembled cucumber genome.

## Conclusions

A genome project requires continual refinement, even after the publication of its genome sequence. Some problems, such as bacterial DNA contamination during genome sequencing and redundancy of repetitive DNA sequences, were found in the first assembly of the cucumber genome; therefore, we reassembled the cucumber genome. After RNA-Seq evidence of transcription was generated, we improved the prediction of protein-coding genes in the reassembled cucumber genome, based upon the RNA-Seq reads. In the new assembly, about 8, 700 protein-coding gene structures are modified and about 5, 200 genes are newly predicted. Based upon the comparison of the gene sets of the two versions, we conclude that the considerable improvement in protein-coding gene prediction is largely due to the use of the RNA-Seq technique. We also suggest that, for newly sequenced or less-studied eukaryotic genomes, RNA-Seq is a good choice for providing evidence for prediction of protein-coding genes, as it reduces the necessity for EST sequencing and increases the utility of each round of genome annotation.

## Methods

### Genome reassembly

To link more contigs, we sequenced additional long insert sized (5 kb) paired-end Illumina GAII reads of *Cucumis sativus *var. *sativus*, representing approximately 7.4-fold genome coverage.

We first assembled paired-end short reads with short insert sizes (insert size < 1 kb) into contigs. To increase the assembly accuracy, only high quality reads were considered. These contigs were further linked into scaffolds by paired-end relationships (300-550 bp insert size), mate-pair reads (2-10 kb), fosmid ends (~40 kb), and BAC ends (~100 kb). We then filled gaps in all the reads generated by both Illumina GAII and Sanger methods.

During the process of linking contigs to scaffolds, paired-end reads with long insert sizes (approximately 3.8-fold genome coverage, 5 Kb insert size; approximately 3.2-fold, 10 Kb insert size) from wild cucumber (*Cucumis sativus *var. *hardwickii*) were also used.

### RNA-Seq experiment

*Cucumis sativus *var. *sativus *line 9930 was used in all experiments. A total of 10 tissues were collected: root, stem, leaf, male flower, female flower, ovary, expanded ovary under fertilization (7 days after flowering), expanded ovary not fertilized (7 days after flowering), base part of tendril, and tendril. In accordance with the manufacturer's instructions, total RNA was isolated with TRIzol (Invitrogen, Carlsbad, CA, USA) from each sample. Samples were treated with RNase-free DNase I for 30 minutes at 37°C (New England BioLabs, Ipswich, MA, USA) to remove residual DNA. The OligoTex mRNA mini kit (QIAGEN, Hilden, Germany) was used to isolate poly(A) mRNA from the total RNA samples. The first cDNA strand was synthesized using random hexamer primers and reverse transcriptase (Invitrogen). The second strand cDNA was synthesized using RNase H (Invitrogen) and DNA polymerase I (New England BioLabs). The sequencing library was constructed following the manufacturer's instructions (Illumina, San Diego, CA, USA). Fragments of approximately 200 bp were excised and enriched by 18 cycles of PCR. The fragments were loaded onto flow cell channels at a concentration of 2 pM to generate paired-end reads with lengths of 75 bp. The Illumina GA processing pipeline v0.2.2.6 was used for image analysis and base calling. The data is obtainable with the accession number SRA046916 in the Sequence Read Archive (SRA) at NCBI.

### Reconstructing transcripts

*De novo *assembly was carried out by Inchworm [19], which utilizes the Kmer graph method to assemble Illumina RNA-Seq reads. Although it prefers strand-specific RNA-Seq reads, Inchworm can also deal with the non-strand-specific RNA-Seq reads generated from the RNA-Seq experiments. Low-coverage artifacts or redundancies from different tissues were removed by CD-HIT [20], with an identity threshold of 95%.

In the 'align-then-assemble' approach, we firstly mapped the RNA-Seq reads from each tissue to the reassembled cucumber genome using Bowtie [21] and the spliced aligner TopHat [22]. Cufflinks [13] assembled the results of TopHat into transcript assemblies, followed by the integration of transcript assemblies from different tissues. Transcripts that were shorter than 150 bp were deemed as false positives and removed before gene prediction. We used *getorf *in EMBOSS [23] to find ORFs in the transcripts. Only ORFs with start and stop codons were regarded as complete ORFs.

### Genome reannotation

RepeatMasker masked the repeat elements in the newly assembled genome using a custom library. The custom library included: (i) Repbase [31]; (ii) TIGR plant repeat database [32]; and (iii) a cucumber *de novo *transposable element library built in-house. Three types of *de novo *software, PILER-DF [33], RepeatScout [34], and LTR_Finder [35] were used to predict species-specific transposable element sequences in the cucumber genome. PILER-DF and RepeatScout were used for the repeat sequences in cucumber assembly. Based on the cucumber assembly, full-length LTR retrotransposons were identified using LTR_Finder. We filtered repeat elements belonging to rRNA, satellites, and organellar sequences by BLASTN. Elements belonging to high-copy number genes were filtered by BLASTX searching of UniProt-SwissProt (release 2010_07). After removing redundant repeat elements by all-versus-all BLASTN and manual curation, the *de novo *TE library for cucumber was obtained.

We used spaln [27] and PASA [24] to align 90, 307 cucumber ESTs sequenced by Guo, Zheng and Joung *et al *[36], 260 cucumber FL-cDNAs downloaded from NCBI, and transcripts reconstructed by Inchworm [19]. The result of 'align-then-assemble' procedure was also directly used as transcript evidence. PASA strictly aligns EST or cDNA sequences to the genome and assembles the aligned sequences into transcripts called 'PASA assemblies'. ORFs are found from these PASA assemblies as a training set. We selected genes with complete structures and removed some redundant genes with 70% identity at the amino acid level by CD-HIT [20].

Five *de novo *gene predictors were used on the masked genome. GlimerHMM [37], SNAP [38], and Augustus [25] were trained with the training set generated by PASA; Geneid [26] used the parameter of *Cucumis *spp.; and GeneMark.hmm-ES [39] only used unmasked genomic data and was self-trained.

The dataset used for protein homology alignment included: (i) UniProt-SwissProt plant proteins (release 2010_07); (ii) *Arabidopsis thaliana *proteins (TAIR9, Augustus 2009 release); and (iii) *Oryza sativa *proteins (TIGR Release 5.0, January 2007 release). We used spaln [40], TBLASTN [28] and BLAT [41] to search for nucleotide homology in the cucumber genome. Scipio [42] made use of the BLAT result to identify intron-exon boundaries. Proteins with the highest score in TBLASTN were processed by BLAST2GENE [43] to predict gene structures by GeneWise [44].

EVM, which is an effective automated annotation combiner [17], computed the gene structures for the reassembled genome of cucumber as a weighted consensus of all available evidence obtained above. The gene models generated by EVM were updated by PASA with ESTs and *de novo *assembled transcripts. This process modified exons or gene models, added UTRs, and found alternatively spliced isoforms. Finally, we removed genes encoding proteins with less than 50 amino acids and incomplete genes without start and stop codons. Gene models and the different evidence were visualized by GBrowse [45].

Three non-coding RNA gene predictors were used independently to identify different types of non-coding RNA genes in the cucumber genome. tRNA-SE [46] was used to identify tRNA genes. Snoscan [47] was used to identify C/D-box small nucleolar RNAs. INFERNAL [48] searches against the Rfam [49] database identified miRNAs, small nuclear RNAs, and H/ACA-box small nucleolar RNAs.

### Comparing gene structural prediction

In annotVer 1.0, 26, 882 CDSs were aligned to the reassembled cucumber genome by spaln [27] and gene structures that have less than 50 amino acids or without start and/or stop codons were removed.

During the comparison, only the coding regions were considered, because the UTRs had more changes between the two versions. Genes with at least one base pair overlapping the coding region were assumed to occupy the same gene locus. If genes occupying the same gene locus had different structures in all alternative spliced isoforms, they were viewed as genes with different structures. We filtered genes with alternative spliced isoforms to simplify further analyses.

When genes mapped to the same locus but with different numbers in the two gene sets (i.e. genes that were merged/split into one or more genes in the other version), we grouped each locus as a group. We then used BLASTP [28] to search each group against UniProt plant proteins (release 2010_07). A group was treated as false positive when no hits were found in UniProt. If a merged gene in annotVer 2.0 and more than two of its counterparts in annotVer 1.0 had the same best hit, the merged event was regarded to be optimal. On the other hand, if the split genes in annotVer 2.0 had more than one best hit, the split structures were considered to be better than the merged structure in annotVer 1.0. In exceptional cases, where only one of the split genes has a best hit, we were confused as to which was better, because there are two conditions we have to consider. If the best hit was longer than the aligned split gene without connection to other split ones, the merged gene would not seem to be better than the split ones. On the other hand, if the best hit was as long as the aligned split gene without other hits to the remaining split ones, then the split genes would also not seem to be better than the merged one.

If different structural genes in the two versions were mapped to the same locus, methods developed by Lorenzi, Puiu and Miller *et al. *[50] were modified and used to describe the structural changes. First, we searched the Pfam domain for each pair of genes with different structures by InterProScan [29]. Then, we used the proteins of each pair to search UniProt plant proteins (release 2010_07). The proteins in the pairs with the same best hit were aligned to the matching proteins in UniProt by *stretcher *in EMBOSS [23]. Gene structures with higher identity, similarity, score, and fewer gaps were considered as better structures.

### Comparing non-overlapped locus protein-coding genes

Comparison of non-overlapped locus protein-coding genes in the two versions was carried out by BLASTP [28] searches against UniProt plant proteins (release 2010_07), with an E value threshold of 10^-5^. The percentage of multi-exon genes was also used as an index to evaluate the gene set for the inaccuracy of single-exon gene prediction.

### Validation by experimental study of WRKY gene family

A dataset of 33 cucumber *WRKY *genes and 35 assembled *WRKY *gene cDNAs generated in a previous experimental study [30] were aligned to the reassembled cucumber genome by spaln [27], followed by manual checking of the differences between the alignment results and gene predictions in annotVer 1.0 and annotVer 2.0.

## Authors' contributions

ZL was responsible for the development and construction of the annotation pipeline and the drafting of the manuscript, while ZZ participated in the reassembly of the cucumber genome and the drafting of the manuscript. PY participated in the RNA-Seq analyses. SH and ZF provided essential suggestions for this work. KL designed and coordinated the work and helped to draft the manuscript. All authors read and approved the final manuscript.

## Supplementary Material

Additional file 1 **Supplemental Tables and Figures**. Table S1. Summary of the additional sequencing data from *Cucumis sativus *var. *hardwickii *and domestic *Cucumis sativus *var. *sativus *to reassembled the genome of *Cucumis sativus *var. *sativus*. Table S2. Statistics of cucumber genome ressembly. Table S3. Mapping RNA-seq reads onto the reassembled cucumber genome. Table S4. Prediction of non-coding RNAs in the two annotations. Table S5. Prediction and classification of transposable elements in the two annotations. Figure S1. Genes in annotVer 1.0 mapped to the same locus of the reassembly of cucumber genome. Figure S2. Two genes in annotVer 1.0 merged into one gene in annotVer 2.0. Figure S3. Genes in annotVer 1.0 and annotVer 2.0 mapped to the same locus but with different structures. Click here for file
