# Supplementary material for: RNA-Seq improves annotation of protein-coding genes in the cucumber genome
Source: BMC Genomics. 2011 Nov 2;12:540. doi: 10.1186/1471-2164-12-540 (PMC3219749; doi:10.1186/1471-2164-12-540)

## Table S1 - Summary of the additional sequencing data from *Cucumis sativus* var. *hardwickii* and domestic *Cucumis sativus* var. *sativus* to reassembled the genome of *Cucumis sativus* var. *sativus*.

| Sequence Data | Insert Size (Kb) | Total Data (Gb) | Sequence Depth (X) | Reads Length (bp) |
| --- | --- | --- | --- | --- |
| *Cucumis sativus* var. *hardwickii* | 5 | 1.40 | 3.82 | 44 |
|  | 10 | 1.16 | 3.17 | 44 |
| *Cucumis sativus* var. *sativus* | 5 | 2.67 | 7.42 | 37 |

## Table S2 - Statistics of cucumber genome reassembly.

|  | **Contig** | | **Scaffold** | | **Super-Scaffold** | |
| --- | --- | --- | --- | --- | --- | --- |
|  | **(bp)** | **Number** | **(bp)** | **Number** | **(bp)** | **Number** |
| N90 | 8,911 | 5,344 | 97,346 | 456 | 281,347 | 153 |
| N80 | 16,122 | 3,757 | 176,676 | 309 | 522,498 | 103 |
| N70 | 22,988 | 2,762 | 254,933 | 216 | 894,084 | 75 |
| N60 | 30,093 | 2,028 | 362,959 | 152 | 1,181,150 | 56 |
| N50 | 37,901 | 1,458 | 488,197 | 105 | 1,407,923 | 40 |

## Table S3 - Mapping RNA-seq reads onto the reassembled cucumber genome.

| **Tissue** | **#Total reads** | **# Paired reads** | **# Mapped reads** | **# Junction reads** | **# Junction** |
| --- | --- | --- | --- | --- | --- |
| *Ovary* | 19,247,768 | 10,709,548 (55.6%) | 17,656,392 (91.7%) | 4,789,671 (24.9%) | 131,898 |
| *Fertilized ovary* | 18,466,067 | 11,177,609 (60.5%) | 17,047,763 (92.3%) | 4,722,299 (25.6%) | 137,086 |
| *Unfertilized ovary* | 19,111,746 | 10,724,534 (56.1%) | 17,394,685 (91.0%) | 4,587,930 (24.0%) | 138,901 |
| *Root* | 18,732,466 | 10,655,725 (56.9%) | 17,162,238 (91.6%) | 4,586,489 (24.5%) | 135,959 |
| *Stem* | 24,535,215 | 15,016,650 (61.2%) | 22,789,659 (92.9%) | 6,668,976 (27.2%) | 138,241 |
| *Leaf* | 26,400,675 | 16,697,721 (63.2%) | 24,405,569 (92.4%) | 6,916,312 (26.2%) | 142,377 |
| *Male flower* | 26,050,858 | 16,826,165 (64.6%) | 24,531,662 (94.2%) | 6,795,276 (26.1%) | 142,571 |
| *Female flower* | 23,818,868 | 14,307,539 (60.1%) | 21,886,487 (91.9%) | 5,991,077 (25.2%) | 138,702 |
| *Tendril* | 22,472,146 | 13,177,475 (58.6%) | 20,585,234 (91.6%) | 6,075,090 (27.0%) | 124,339 |
| Base part of tendril | 21,653,855 | 13,015,399 (60.1%) | 19,556,866 (90.3%) | 5,636,150 (26.0%) | 116,043 |

## Table S4 – Prediction of non-coding RNAs in the two annotations.

| **RNA type** | **annotVer 2.0** | **annotVer 1.0** |
| --- | --- | --- |
| rRNA | 20 | 41 |
| CD-snoRNA | 122 | 140 |
| HACA-snoRNA | 79 | 45 |
| miRNA | 1,025 | 1,074 |
| snRNA | 157 | 154 |
| tRNA | 621 | 691 |
| Total | 2,024 | 2,145 |

## Table S5 – Prediction and classification of transposable elements in the two annotations.

| **Classification** | **annotVer 2.0** | | | **annotVer 1.0** | | |
| --- | --- | --- | --- | --- | --- | --- |
|  | **Elements number** | **Total length (bp)** | **Mask ratio (%)** | **Elements number** | **Total length (bp)** | **Mask ratio (%)** |
| DNA Transposons | 16,559 | 2,724,165 | 1.41 | 16,972 | 2,808,075 | 1.24 |
| Retrotransposons | 133,681 | 25,988,002 | 13.42 | 119,339 | 27,538,485 | 12.16 |
| *LTR* | 107,687 | 22,209,388 | 11.47 | 91,109 | 23,622,636 | 10.43 |
| *LINE* | 20,678 | 3,757,553 | 1.94 | 16,899 | 3,937,077 | 1.74 |
| *SINE* | 149 | 8,164 | 0.00 | 195 | 14,911 | 0.01 |
| *Unclassified* | 5,167 | 146,518 | 0.06 | 11,136 | 3,507,231 | 0.01 |
| Unclassified | 67,586 | 13,570,276 | 7.01 | 135,464 | 26,367,990 | 11.64 |
| Total | 217,826 | 40,421,591 | 20.87 | 266,232 | 54,361,644 | 23.01 |

## Figure S1 - Genes in annotVer 1.0 mapped to the same locus of the reassembly of cucumber genome.


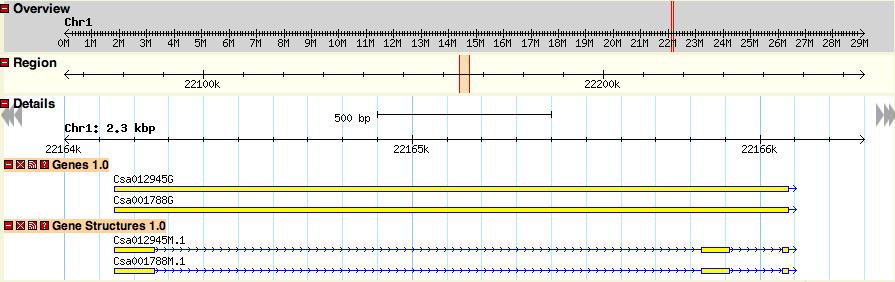


## Figure S2 - Two genes in annotVer 1.0 merged into one gene in annotVer 2.0.


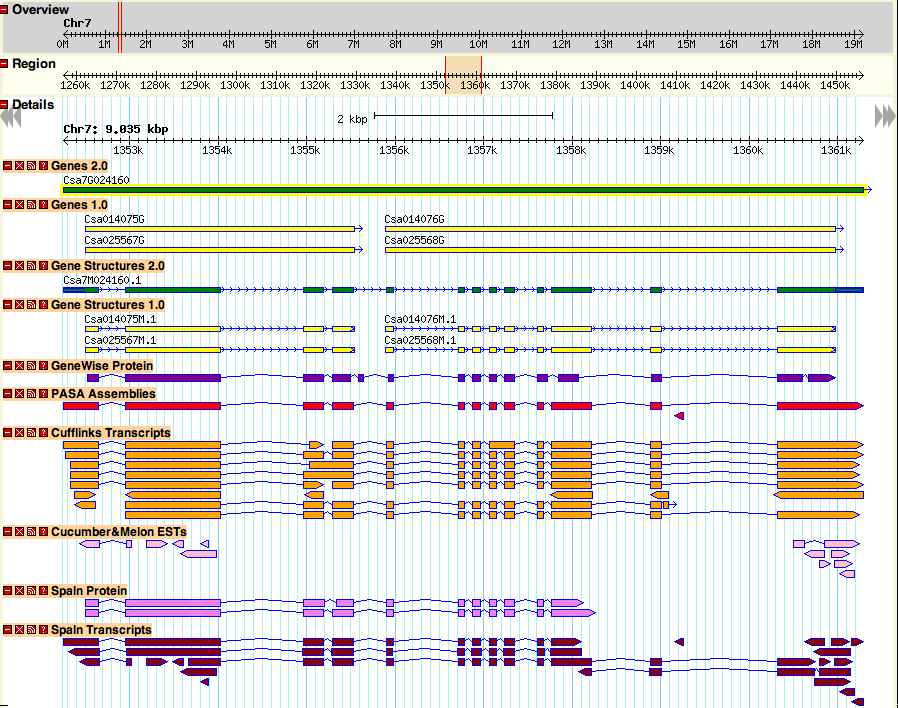


## Figure S3 - Genes in annotVer 1.0 and annotVer 2.0 mapped to the same locus but with different structures.


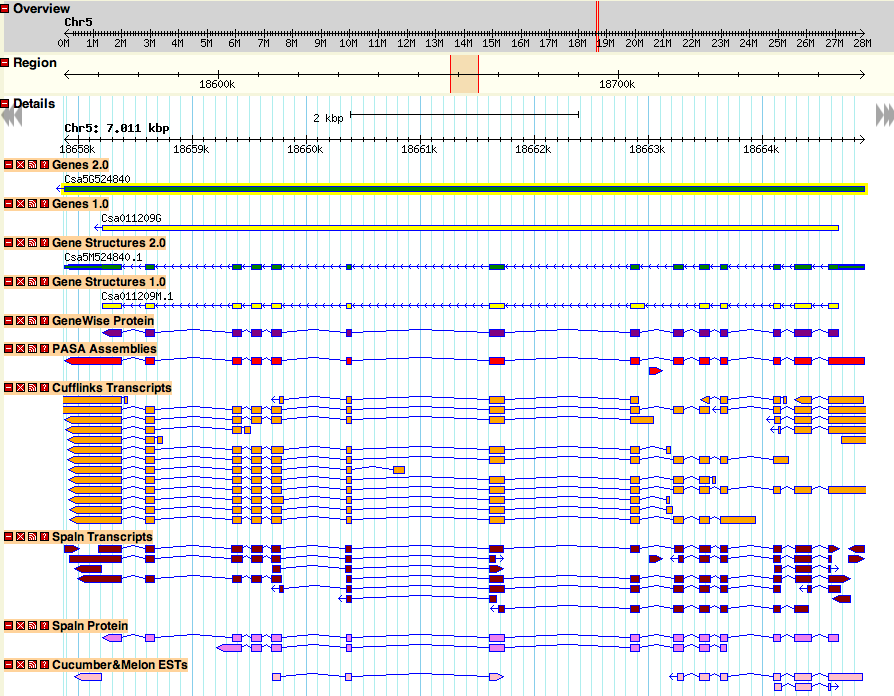

Supplement: Additional file 1 — Supplemental Tables and Figures. Table S1. Summary of the additional sequencing data from Cucumis sativus var. hardwickii and domestic Cucumis sativus var. sativus to reassembled the genome of Cucumis sativus var. sativus. Table S2. Statistics of cucumber genome ressembly. Table S3. Mapping RNA-seq reads onto the reassembled cucumber genome. Table S4. Prediction of non-coding RNAs in the two annotations. Table S5. Prediction and classification of transposable elements in the two annotations. Figure S1. Genes in annotVer 1.0 mapped to the same locus of the reassembly of cucumber genome. Figure S2. Two genes in annotVer 1.0 merged into one gene in annotVer 2.0. Figure S3. Genes in annotVer 1.0 and annotVer 2.0 mapped to the same locus but with different structures. [file 1471-2164-12-540-S1.DOC]
